# Lunar gravity prevents skeletal muscle atrophy but not myofiber type shift in mice

**DOI:** 10.1038/s42003-023-04769-3

**Published:** 2023-04-21

**Authors:** Takuto Hayashi, Ryo Fujita, Risa Okada, Michito Hamada, Riku Suzuki, Sayaka Fuseya, James Leckey, Maho Kanai, Yuri Inoue, Shunya Sadaki, Ayano Nakamura, Yui Okamura, Chikara Abe, Hironobu Morita, Tatsuya Aiba, Teruhiro Senkoji, Michihiko Shimomura, Maki Okada, Daisuke Kamimura, Akane Yumoto, Masafumi Muratani, Takashi Kudo, Dai Shiba, Satoru Takahashi

**Affiliations:** 1 Laboratory Animal Resource Center in Transborder Medical Research Center, and Department of Anatomy and Embryology, Institute of Medicine, University of Tsukuba, Ibaraki, 305-8575 Japan; 2 Doctoral Program in Biomedical Sciences, Graduate School of Comprehensive Human Sciences, University of Tsukuba, Ibaraki, 305-8575 Japan; 3 Divsion of Regenerative Medicine, Transborder Medical Research Center, Institute of Medicine, University of Tsukuba, Ibaraki, 305-8575 Japan; 4 JEM Utilization Center, Human Spaceflight Technology Directorate, Japan Aerospace Exploration Agency (JAXA), Ibaraki, 305-8505 Japan; 5 Mouse Epigenetics Project, ISS/Kibo experiment, JAXA, Ibaraki, 305-8505 Japan; 6 Ph.D. Program in Human Biology, School of Integrative and Global Majors, University of Tsukuba, Ibaraki, 305-8575 Japan; 7 Ph.D. Program in Humanics, School of Integrative and Global Majors, University of Tsukuba, Ibaraki, 305-8575 Japan; 8 College of Medicine, School of Medicine and Health Sciences, University of Tsukuba, Ibaraki, 305-8575 Japan; 9 Department of Physiology, Gifu University Graduate School of Medicine, Gifu, 501-1194 Japan; 10 Department of Nutrition Management, Tokai Gakuin University, Gifu, 504-8511 Japan; 11 Department of Genome Biology, Transborder Medical Research Center, Institute of Medicine, University of Tsukuba, Ibaraki, 305-8575 Japan

**Keywords:** Transcriptomics, Molecular medicine

## Abstract

Skeletal muscle is sensitive to gravitational alterations. We recently developed a multiple artificial-gravity research system (MARS), which can generate gravity ranging from microgravity to Earth gravity (1 *g*) in space. Using the MARS, we studied the effects of three different gravitational levels (microgravity, lunar gravity [1/6 *g*], and 1 *g*) on the skeletal muscle mass and myofiber constitution in mice. All mice survived and returned to Earth, and skeletal muscle was collected two days after landing. We observed that microgravity-induced soleus muscle atrophy was prevented by lunar gravity. However, lunar gravity failed to prevent the slow-to-fast myofiber transition in the soleus muscle in space. These results suggest that lunar gravity is enough to maintain proteostasis, but a greater gravitational force is required to prevent the myofiber type transition. Our study proposes that different gravitational thresholds may be required for skeletal muscle adaptation.

## Abstract (web summary)

Artificial lunar gravity is shown to be sufficient to suppress microgravity-induced soleus muscle atrophy but not myofiber type shift in mice.

## Introduction

The gravity on Earth plays an essential role in the development of organisms, including human beings. However, the mechanisms through which gravity regulates cellular and molecular processes, such as gene expression, which influence the structure and function of tissues and organisms remain poorly understood. Considering the future human space exploration and colonization programs, such as the Artemis mission (National Aeronautics and Space Administration [NASA] human lunar exploration plan for 2024), understating how gravity affects the molecular biological systems might serve as new foundation for humankind further space exploration.

Skeletal muscle is the most abundant tissue in the body, representing more than 40% of the total body mass. This tissue shows remarkable plasticity in volume, contractile function, and energy metabolism. Skeletal muscle adapts in response to mechanical demands, indicating the presence of mechanosensitive receptors^1–4^. Mechanical overloading, such as that which occurs during resistance training, can result in muscle hypertrophy. In addition to high mechanical demands, mechanical force in the form of gravity constantly also acts on the skeletal muscle^5^. Therefore, prolonged periods of microgravity exposure, during space flight or bedrest, rapidly reduce skeletal muscle mass and strength^5^. Skeletal muscle atrophy results from decreased protein synthesis and increased protein degradation, and long-term muscle wasting is a major risk factor for various diseases and increased mortality^6–8^. The major anabolic pathway in skeletal muscle is primarily regulated by insulin and the insulin-like growth factor (IGF)–Akt–mTOR pathway^9,10^. Under catabolic conditions, occurs the breakdown of the muscle structural proteins and organelles through major cellular protein degradation systems, namely, the transcription-dependent induction of the ubiquitin‐proteasome and autophagy‐lysosome pathways^11^. However, it remains unclear how the skeletal muscle regulates proteostasis in response to mechanical signals, such as shifts in gravity. Understanding this process is great translational importance, especially for space exploration.

Skeletal muscle comprises a mosaic myofiber with different contractile and metabolic properties^12^. Slow-twitch myofibers express high levels of type I myosin heavy chain (MHC) and are rich in mitochondria, which are determinant for oxidative phosphorylation and ATP production^12^. In contrast, fast-twitch myofibers express three subtypes of type II MHCs: IIa, IIx, and IIb. Type IIb myofibers exhibit low mitochondrial density and mostly rely on anaerobic glycolysis for energy production. Type IIa/x myofibers exhibit hybrid characteristics between type I and type IIb fibers. Skeletal muscle myofiber types dynamically shift during mechanical loading or unloading^13^. Unloading, which occurs under conditions of microgravity or disuse, stimulates slow-to-fast transition in type I myofibers, including those in the soleus (Sol) muscle. Skeletal muscle myofiber transition is regulated by several factors, such as peroxisome proliferator-activated receptor (PPAR)-γ-coactivator-1α (PGC-1α), PGC-1β^14,15^, NFATc1^16^, Baf60c^17^, and oxidative stress^18^. PGC-1α is the most well-described factor and a powerful regulator of slow-oxidative myofiber formation. The main driver for fast-glycolytic myofiber formation has not yet been completely determined^19–22^. Furthermore, the role of mechanical stress, including gravity, in skeletal muscle myofiber-type transition remains unclear^23^.

Several studies have investigated the effects of gravity on skeletal muscle adaptation at the International Space Station (ISS)^18,24–26^. Our group recently developed a multiple artificial-gravity research system (MARS) that employs a centrifuge to create artificial gravity, enabling comparative studies between the effects of gravity and microgravity in space^27,28^. Studies using this system revealed skeletal muscle weight loss and slow-to-fast myofiber shifts after 35 days in microgravity space flight; however, these effects are completely inhibited under artificial gravity of 1 *g*. These results demonstrated that gravity, not radiation or other stress, is the primary regulator of skeletal muscle homeostasis in space flight^26^. However, the gravitational threshold required to maintain skeletal muscle weight and myofiber composition is unknown.

In this study, we sought to understand whether lunar gravity (1/6 *g*) is strong enough to prevent changes in skeletal muscle adaptation during space flight. Using the developed MARS, we demonstrated that 1/6 *g* can prevent microgravity-induced atrophy in the Sol muscle. However, the slow-to-fast myofiber transition of the Sol muscle occurring during microgravity was not suppressed at 1/6 *g*. These results suggest that muscle mass and myofiber type are regulated at different gravitational thresholds. Our study is the first to suggest the existence of a gravitational threshold for skeletal muscle adaptation in mammals and will serve as a pilot study for elucidating the effect of gravity on living organisms.

## Results

### Overview of the mouse habitat unit (MHU) missions

**Fig. 1:**
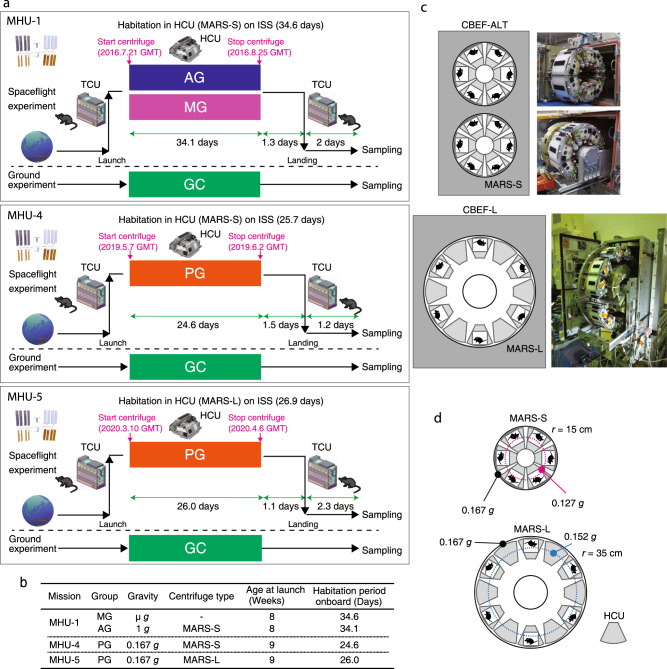
Overview of space flight experiments after return to Earth. **a** Overview of mouse habitat unit (MHU) missions using the multiple artificial-gravity research system (MARS). C57BL/6 J male mice (8 or 9 weeks old) were launched from the Kennedy Space Center using a transportation cage unit (TCU). On the International Space Station (ISS), the mice were transferred into habitat cage units (HCUs) installed in the MARS-S or MARS-L for gravity loading. After onboard housing for certain periods, mice were transferred back to the TCU and landed on the West Coast of the USA. **b** Basic information on each experimental group of space mice in the three missions. MG microgravity, AG artificial gravity (1 *g*), PG partial gravity (1/6 *g*). **c** Multiple artificial-gravity research system (MARS) platform on the International Space Station (ISS). Top: Centrifuge-equipped biological experiment facility (CBEF-ALT). The CBEF-ALT has two compartments: the microgravity section (upper) and the artificial gravity section (lower) with a centrifuge. Bottom: Artificial gravity section with a large centrifuge. **d** Characteristics of the two types of centrifugal devices used, MARS-S and MARS-L. The magenta and blue dotted lines indicate the position of the mouse head on each device.

To elucidate the effect of different gravitational forces (microgravity, 1/6 *g*, and 1 *g*) in skeletal muscles of mice during space flight, we conducted three space missions, MHU-1, MHU-4, and MHU-5, in which male C57BL/6 J mice (8 or 9 weeks old) were maintained on the ISS for approximately one month (Fig. 1a, b). In MHU-1, microgravity and 1 *g* artificial gravity were compared using the MARS with a short radius centrifuge (MARS-S), and various tissues were evaluated to determine the influence of gravity^28^. In MHU-4 and – 5, gravity was set to 1/6 *g* (0.167 *g*), to mimic the gravity of the moon, as the most feasible future missions are to explore the moon. For MHU-4 and – 5, HCUs were placed on the floor, each housing six mice, and a short radius centrifuge (MARS-S) and a long radius centrifuge (MARS-L), respectively, were used to generate 1/6 *g* (Fig. 1c, d). We analyzed the skeletal muscle of mice housed in space for a month with different gravitational loads (microgravity, MG; partial gravity [1/6 *g*], PG; artificial gravity [1 *g*], AG) and on earth (ground control, GC). The environmental parameters and habitation data are summarized in Supplementary Information 1, Supplementary Tables 1 and 2. In each mission, the health of each mouse was monitored daily by veterinarians on the ground via a downlinked video system^29^. Three representative mice from each group are shown in Supplementary Movie 1. Forelimbs and hindlimbs were contacted to the bottom of habitation cage in both 1 *g* and 1/6 *g* conditions (MHU-4 and MHU-5). After the space missions, upon return to Earth, mice showed no apparent abnormalities based on the condition of the eyes, ears, teeth, fur, and tail. In MHU-5, three mice showed significant weight loss after flight due to issues with the water nozzle in the transportation cage unit (TCU) (Supplementary Fig. 1a, b) and were excluded from further data analysis. The extent of weight change in the PG group compared to that in the GC group was similar between MHU-4 and MHU-5 (Supplementary Fig. 1c). The ground test using the TCU used for live animal return from the ISS reproduced this body weight loss of mice reared in cages that the weight loss was observed (Supplementary Fig. 2)

### Lunar gravity prevents skeletal muscle atrophy during space flight

**Fig. 2:**
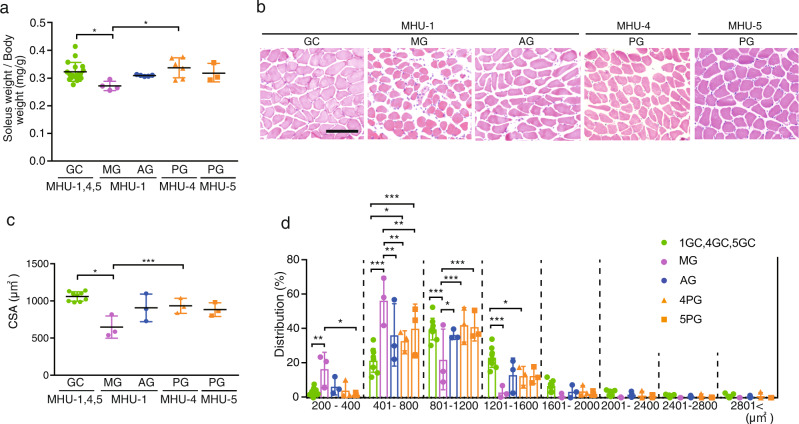
Effect of lunar gravity on the soleus muscle. **a** Soleus muscle weights normalized to total body weight (mg/g) from mouse habitat unit (MHU)-1, -4, and -5 missions and ground control (GC). Data are represented as the mean and standard deviation (SD), and each point represents an individual mouse; MHU-1_MG: *n* = 4; MHU-1_AG: *n* = 5; MHU-4_PG: *n* = 6; MHU-5_PG: *n* = 3; GC: *n* = 18. **b** Hematoxylin–eosin staining of the soleus muscle cross sections. Scale bar, 100 μm. **c** Cross-sectional area (CSA) of the soleus myofibers in each group (*n* = 3). *n* = 9 for GC. Data are represented as the mean and SD, and each point represents an individual mouse. **d** CSA distribution of the soleus muscles in each group (*n* = 3). *n* = 9 for GC. Data are represented as the mean and SD, and each point represents an individual mouse. ^*^P < 0.05, ^**^P < 0.01, and ^***^P < 0.001, as determined using Tukey’s test. 1GC: MHU-1_GC; 4GC: MHU-4_GC; 5GC: MHU-5_GC; MG: MHU-1_MG; AG: MHU-1_AG; 4PG: MHU-4_PG; 5PG: MHU-5_PG.

We first examined the effect of different gravitational levels on the skeletal muscle mass of the Sol, plantaris (Pla), gastrocnemius (Gas), tibialis anterior (TA), and extensor digitorum longus (EDL) muscles. The Sol muscle, composed of slow-oxidative type I/IIa myofibers, is highly susceptible to microgravity and exhibits significant atrophy in space^30–32^. The wet weight of the Sol muscle, normalized to the body weight, was 17% lower in the MG group than in the GC group but was similar between the GC and PG groups of MHU-4 and MHU-5 (Fig. 2a). Similarly, the Pla muscle wet weight was 12% lower in the MG group than in the GC group; however, this difference diminished in the PG group of MHU-4, but not in the PG group of MHU-5 (Supplementary Fig. 3). Normalized Gas muscle wet weight was significantly lower in the MG and PG groups than in the GC group. TA muscle wet weight was reduced by 12% in the PG group of MHU-5 compared to that in the AG group, but no differences were observed between the MG and PG groups of MHU-4. Furthermore, EDL muscle weight was reduced by 12% in the PG group of MHU-4 compared to that in the GC group, but no differences were observed between the MG and PG groups of MHU-5.

We performed histological analysis on the Sol and EDL muscles, which are predominantly composed of slow- and fast-twitch myofibers, respectively. Hematoxylin–eosin (HE) staining showed no abnormalities, such as central nucleation or inflammatory cell infiltration, in any group (Fig. 2b and Supplementary Fig. 4a). Cross-sectional area (CSA) analysis of Sol muscles revealed a 40% reduction in myofiber size in the MG group, but not in the AG or PG groups (Fig. 2c, d). CSA analysis of the EDL muscle did not reveal any differences among groups (Supplementary Fig. 4b, c). These results suggest that 1/6 *g* can prevent atrophy in the Sol muscles during space flight.

### Gene expression of the skeletal muscle is altered under different gravitational loads

**Fig. 3:**
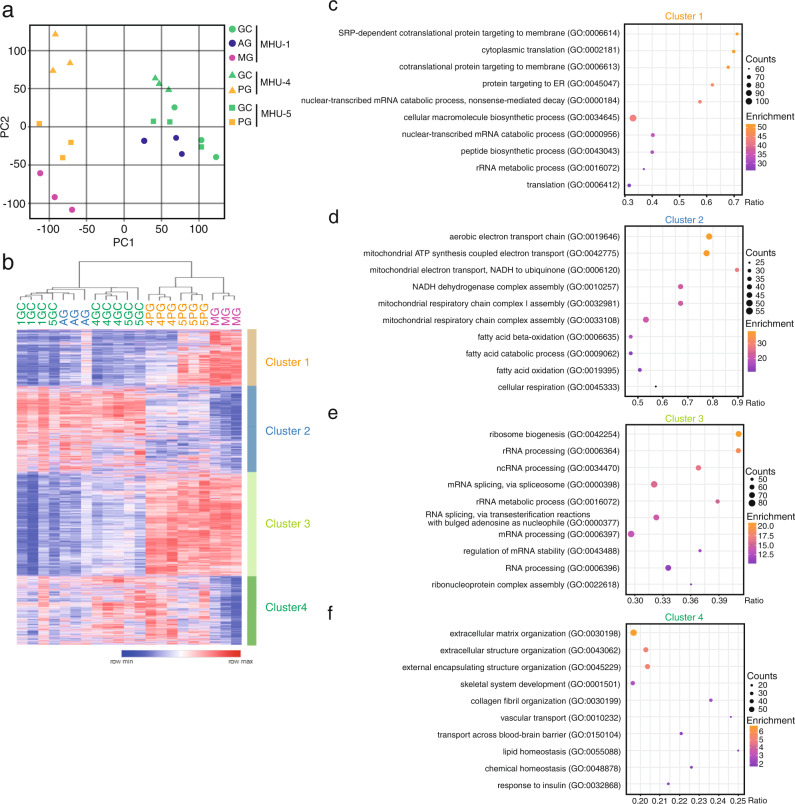
Comprehensive RNA sequencing analysis under conditions of microgravity and lunar gravity. **a** Principal component (PC) analysis plots of gene expression of the soleus muscle of mice in mouse habitat unit (MHU)-1, -4, and -5 missions. **b** Clustering heatmap of 7138 differentially expressed genes between MHU-1_MG vs. MHU-1_GC. 1GC: MHU-1_GC; 4GC: MHU-4_GC; 5GC: MHU-5_GC; MG: MHU-1_MG; AG: MHU-1_AG; 4PG: MHU-4_PG; 5PG: MHU-5_PG. **c**–**f** Gene Ontology analysis of each gene cluster.

To determine how gravity affects gene expression in skeletal muscle, we performed RNA sequencing (RNA-seq) analysis of the Sol muscles. Principal component analysis (PCA) showed similar transcriptomes between AG and GC mice. Contrarily, PG transcriptome showed more similarities to MG than to GC or AG mice. However, the most striking distance was between the PG and MG transcriptomes (Fig. 3a). These results suggest that the expression of some genes was regulated by gravity above 1/6 *g*. Next, we analyzed gene expression in detail to reveal the unique gene expression patterns under distinct gravitational conditions. A total of 7138 genes with a false discovery rate (FDR) < 0.05 were visualized by cluster heat mapping between MG and GC mice, revealing four distinct gene clusters (Fig. 3b). Clusters 1 and 2 were up- and downregulated, respectively, in MG mice compared to that in GC mice. Gene Ontology (GO) analysis revealed that clusters 1 and 2 contained genes mostly involved in RNA metabolism and oxidative phosphorylation, respectively (Fig. 3c, d). Cluster 3, upregulated in MG and PG mice, included genes related to the ribosome biosynthesis pathway, suggesting an involvement in skeletal muscle growth or mass (Fig. 3e). Cluster 4, downregulated only in MG mice, comprised genes associated with extracellular matrix (ECM) organization, which ensures mechanical connection in the skeletal muscle (Fig. 3f).

**Fig. 4:**
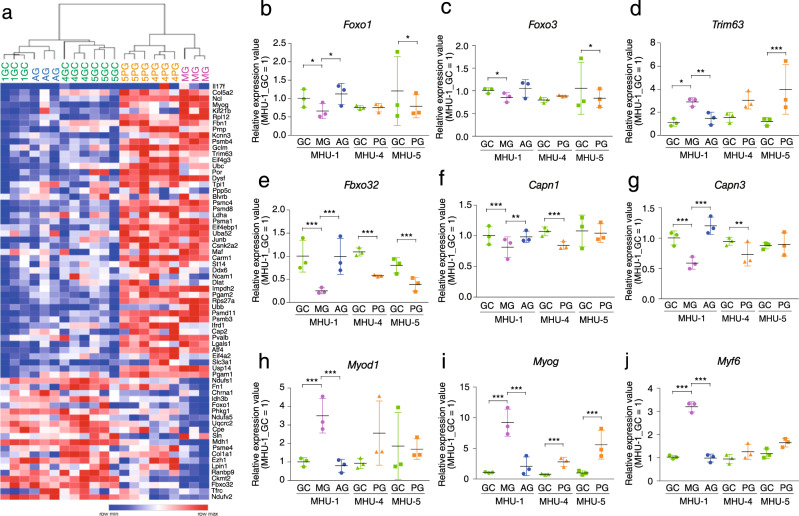
Analysis of genes related to skeletal muscle plasticity under conditions of microgravity and lunar gravity. **a** Heatmap of atrogene expression values in the soleus muscles from mouse habitat unit (MHU)-1, -4, and -5_GC, MHU-1_MG, MHU-1_AG, MHU-4_PG, and MHU-5_PG mice. 1GC: MHU-1_GC; 4GC: MHU-4_GC; 5GC: MHU-5_GC; MG: MHU-1_MG; AG: MHU-1_AG; 4PG: MHU-4_PG; 5PG: MHU-5_PG. **b**–**j** Gene expression of *Foxo1*, *Foxo3*, *Trim63*, *Fbxo32*, *Capn1*, *Capn3*, *Myod1*, *Myog*, and *Myf6* in the soleus muscle. Data are represented as the mean and SD, and each point represents an individual mouse (*n* = 3). Expression values are normalized by transcripts per million (each MHU-1_GC = 1). *P* values were calculated using the edgeR test; ^*^P < 0.05, ^**^P < 0.01, and ^***^P < 0.001 for all groups in panels **b**–**j** (false discovery rate-corrected).

Skeletal muscle atrophy can activate common atrophy-related genes, referred to as atrogenes. Heat mapping of atrogenes demonstrated a similar expression pattern in PG and MG mice compared to that in GC mice (Fig. 4a). We then compared the expression of *Foxo1*, *Foxo3*, *Fbxo32* (atrogin*-1*), and *Trim63* (*MuRF1*), which is upregulated in response to skeletal muscle atrophy, including cachexia, denervation, and disuse muscle atrophy^33,34^. We also compared the expression of the Ca^2+^‑dependent cysteine proteases, *Capn1* and *Capn3*, as recessive mutations in these genes cause muscular dystrophy^35,36^. No significant differences were found in the gene expression profiles between MG and PG mice in any mission (Fig. 4b–g), despite the reduced muscle atrophy observed in PG mice (Fig. 2b–d). We also analyzed the expression of the myogenic regulators *Myod1*, *Myog*, and *Myf6*—transcriptional factors for skeletal muscle development and differentiation^37–42^. The expression of these genes was significantly higher in MG than in GC mice; however, this upregulation was suppressed in PG and AG mice (Fig. 4h–j). These results might derive from the dual role of myogenin—a regulator of myogenesis and an inducer of neurogenic muscle atrophy^43^. Together, these data suggest that atrogene expression is not deeply affected by the 1/6 *g* environment and that the suppression of myogenic regulatory factors (MRFs) could be responsible for the inhibitory effect of PG and AG against muscle atrophy in space.

### Microgravity-induced slow-to-fast myofiber transition is not prevented by lunar gravity

**Fig. 5:**
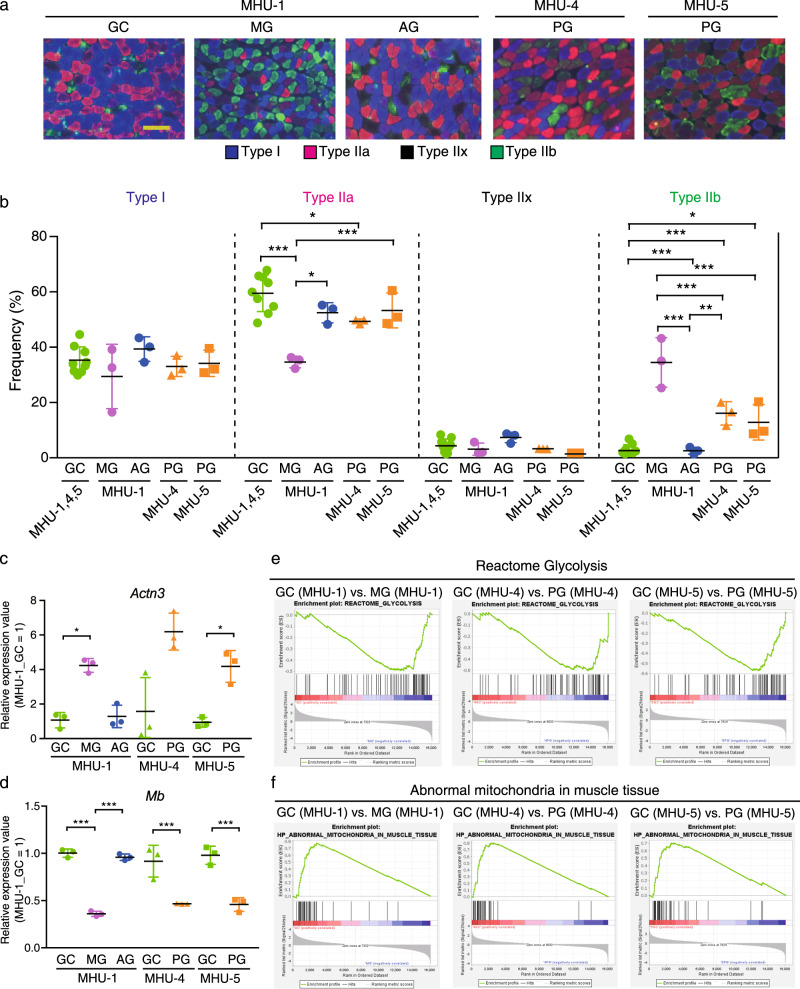
Effect of lunar gravity on myofiber type in the soleus muscle. **a** Immunohistochemical staining for myosin heavy chain using BA-D5 (type I; blue), SC-71 (type IIa; red), and BF-F3 (type IIb; green) antibodies. Unstained myofibers are defined as type IIx (black). Scale bar, 100 μm. **b** Frequency of each myofiber type in all groups. Data are represented as the mean and SD, and each point represents an individual mouse; *n* = 9 for GC and *n* = 3 for all other groups. ^*^P < 0.05, ^**^P < 0.01, and ^***^P < 0.001, as determined using Tukey’s tests. **c** *Actn3* expression in the soleus muscle. **d** *Mb* expression in the soleus muscle. Expression values are normalized by transcripts per million (each GC = 1). Data are represented as the mean and SD, and each point represents an individual mouse; *n* = 3 for each group. ^*^P < 0.05, ^**^P < 0.01, and ^***^P < 0.001 (false discovery rate-corrected), as determined using the edgeR test (**c**, **d**). **e** Gene set enrichment analysis (GSEA) of “Reactome Glycolysis” for each mouse habitat unit (MHU) mission. **f** GSEA of “Abnormal mitochondria in muscle tissue” for each MHU mission.

Microgravity induces slow-to-fast myofiber-type transition in the Sol muscle^44^. Considering that Sol muscle atrophy was partially suppressed by lunar gravity (Fig. 2b–d), we investigated whether 1/6 *g* was sufficient to prevent microgravity-induced slow-to-fast myofiber transition. Cross sections of the Sol and EDL muscles were immunostained with antibodies against type I, IIa, and IIb. Fibers not identified as type I, IIa, or IIb were defined as type IIx.” (Fig. 5a, Supplementary Fig. 5a). The percentage of type IIa and IIb myofibers in the Sol muscle was 43% lower and 1000% higher, respectively, in MG mice than in GC mice (Fig. 5b), indicating slow-to-fast myofiber transition during space flight. These changes were competently abolished in AG mice. The altered IIa:IIb ratio in MG mice was partially reverted in PG mice. The EDL muscle myofibers were not affected by space flight (Supplementary Fig. 5b).

To analyze these differences at the transcriptional level, we determined the expression of abundantly expressed genes in slow- or fast-twitch muscles. The expression of *Actn3*, expressed in fast-twitch myofibers^45^, was significantly upregulated in MG mice compared to that in GC mice, and this change was completely suppressed in AG mice. Furthermore, PG mice showed upregulated expression of *Actn3* (Fig. 5c). The expression of myoglobin (*Mb*), expressed in slow-twitch myofibers^46^, was downregulated in MG mice compared to that in GC mice; no differences were observed between GC and AG mice (Fig. 5d); PG mice exhibited downregulated *Mb* expression.

Slow-twitch myofibers require oxidative metabolism for energy, whereas fast-twitch myofibers rely on glycolysis^45^. Therefore, we performed gene set enrichment analysis (GSEA) to demonstrate whether glycolysis-associated pathways were enriched in MG and PG mice at the gene level. The glycolysis gene set was enriched in MG and PG mice compared to that in GC mice (Fig. 5e). In contrast, the gene set associated with mitochondria in muscle tissues was downregulated in MG and PG mice (Fig. 5f). Together, these results suggest that lunar gravity is not sufficient to inhibit microgravity-induced slow-to-fast myofiber type transition in the Sol muscle.

## Discussion

Space travel has always been a great promise for humankind. Currently, NASA is planning human-crewed missions to Mars. Therefore, a better understanding of the effects of gravity on human biology is required. Skeletal muscle is one of the most sensitive tissues to environmental changes, including gravitational alterations, due to its high plasticity to mechanical stress. Therefore, how gravitational force regulates skeletal muscle mass and myofiber type is one of the issues to be clarified for human spaceflight.

We previously developed the MARS on ISS, which can create artificial gravity to study the different gravitational loads in space^27,28^. We first reported that artificial gravity of 1 *g* in space prevents the microgravity-induced changes in mouse skeletal muscle, including the reduction in muscle mass and myofiber-type transition^26^. Additionally, skeletal muscle atrophy in rats can be prevented by artificial gravity of 1 *g* in space^47^. Therefore, we sought to understand the intensity of gravity required to suppress these muscle changes, i.e., if there is a gravity threshold. In this study, we used the MARS to investigate the effect of lunar gravity on skeletal muscle homeostasis during space flight. Microgravity-induced atrophy was suppressed at 1/6 *g* (lunar gravity); however, this was not reflected in changes in the myofiber type. Our results provide the first evidence that skeletal muscle mass and myofiber type change during unloading and are differently regulated at distinct gravitational levels. Furthermore, our RNA-seq data elucidate the effects of different gravitational forces (microgravity, 1/6 *g*, and 1 *g*) on the skeletal muscle, without interference of other relevant factors of space travel, such as radiation or stress.

The gravitational threshold for regulating skeletal muscle mass is lower than that for regulating myofiber type, indicating that gravity greater than 1/6 *g* might be required to prevent slow-to-fast myofiber type transition. This result is supported by our RNA-seq results, which showed that the microgravity-induced upregulated expression of atrogenes, including Trim63^33^ and myogenin^43^, was significantly inhibited in a 1/6 *g* environment. Furthermore, the identified gene cluster 4, downregulated only under microgravity, suggests that ECM-related genes might be involved in microgravity-induced atrophy. ECM localized in the tendon, muscles, and perimusculature ensures a mechanical link between the skeletal muscle and the bone. Force transmission is based on chains of ECM proteins, including integrins, fibronectin, collagen, and vinculin, in the myotendinous junction (MTJ)—the specialized structure between the skeletal muscle and tendon^48^. The MTJ can be modified in response to mechanical stimuli, such as resistance training and tail suspension in rodents^49,50^. The mechanism regulating MTJ activity during loading or unloading remains unclear. However, lunar gravity appears to partially suppress microgravity-induced muscle atrophy by strengthening the cross-linking in the connective tissues between the skeletal muscle and bone.

Muscle size is determined by the balance between protein synthesis and degradation^51^. Loss of muscle mass is regulated by the activation of atrophy-related genes or atrogenes^52^. Here, we analyzed atrogenes, including Foxo1, Fbxo32, and Trim63, Calpain, and myogenin, as they are important for myogenic differentiation and induce denervation-induced atrophy^43^. Our analysis demonstrated that 1 *g* conditions yielded similar expression patterns of *Fbxo32*, *Trim63*, and *Myog* to those seen in GC mice, suggesting that artificial gravity (1 *g*) can prevent microgravity-induced changes in atrogene expression. However, in the MHU-4 and MHU-5 missions, the expression patterns of these atrogenes in the PG group varied, and 1/6 *g* did not significantly affect gene expression, although the reduction in Sol muscle weight and CSA were attenuated. Notably, MuRF1 is a primary regulator of multiple types of muscle atrophy^53^ on Earth but not in space flight-induced atrophy^24^, indicating that atrophy in space induces unique catabolic pathways. Further study of our RNA-seq data is required to identify genes uniquely suppressed in the 1/6 *g* environment.

Clusters 2 and 3 were commonly altered under 1/6 *g* and microgravity, suggesting an association with myofiber-type transition. Cluster 2, which contained genes whose expression was generally downregulated in both MG and PG mice compared to that in the GC group, was associated with pathways related to mitochondria and ATP production (Fig. 3d). Similarly, our immunohistochemical analysis indicated that 1 *g* entirely suppressed the microgravity-induced reduction in type IIa number and elevation in type IIb myofibers number in the Sol muscles. Furthermore, 1 *g* was sufficient to inhibit changes in the mRNA levels of *Actn3* (fast-enriched) and myoglobin (slow-enriched), whereas 1/6 *g* was not. Therefore, we conclude that a gravity threshold exists between 1 and 1/6 *g* that can suppress the slow-to-fast myofiber-type transition induced by microgravity. Future studies using the MARS are warranted to identify the exact gravity threshold and molecular mechanisms required to prevent the myofiber-type transition in space.

Based on the notions of the effect of scaling lows and the differences in myofiber-type proportions across species^54^, the susceptibility to gravity might differ between humans and other species. Despite its merits, our study has some limitations. First is that as our space experiment only used mice, it is unclear whether the present findings can be applied to human biology. Another limitation is that the skeletal muscle samples were collected on the second day after return. Therefore, we cannot exclude the possibility that our data, especially RNA-seq data, may have been altered by the effects of re-loading. Another group performed a similar space experiment (Bion-M 1) comparing the effects of artificial gravity and microgravity^55^, in which the gene expression of Sol muscles snap-frozen within 12–14 h following landing was analyzed using microarray. Our RNA-seq data are consistent with the reported gene expression patterns of MRFs in Bion-M 1. Therefore, we believe that our mRNA profiles of the skeletal muscle under different gravities (microgravity, 1/6 *g*, and 1 *g*) will serve as a resource for future studies on skeletal muscle adaptation in space.

In the present study, we conducted three independent space missions (MHU-1, – 4, and – 5). In the first mission (MHU-1)^26^, the Sol muscle of mice subjected to AG (1 *g* in space) was similar to that of mice subjected to GC (1 *g* on the ground), histologically and transcriptionally. These data led us to conclude that cosmic radiation and other factors from space had little effect on the Sol muscle changes. Therefore, we investigated the gravity threshold that could prevent skeletal muscle atrophy in space as MHU-4 and -5 missions. As we could not include the AG group as a control in MHU-4 and -5, we compared the differences in the gene expression of PG and GC mice in MHU-4 and -5, to minimize the batch effect of RNA-seq between missions. Future space missions should be designed to include AG as a control. In addition, two types of centrifuges were used to generate 1/6 *g* in MHU-4 and -5. Due to the different radius lengths of the centrifuges, the gravitational load to the head position differed by ~0.025 *g* between MHU-4 and -5, which might have induced slight differences in the skeletal muscle weight and RNA-seq results between the MHU-4 and -5 missions.

In conclusion, using the MARS, we found that artificial gravity equivalent to 1/6 *g* is sufficient to suppress microgravity-induced atrophy of the Sol muscle. However, microgravity-induced slow-to-fast myofiber-type transition in the Sol muscle is not prevented by lunar gravity. Our study that reveals the gravitational threshold between skeletal muscle mass and myofiber type using space environment provides a platform for exploring the mechanosensory mechanisms controlling the skeletal muscle response to alterations in gravity. The elucidation of these mechanisms may lead to the development of strategies to maintaining skeletal muscle health during long-term space travel in the future.

## Methods

### Animals

C57BL/6 J male mice (Stock #000664) were purchased from Jackson Laboratories (Bar Harbor, ME, USA) and Charles River Laboratories (Yokohama, Japan) for the MHU-1, – 4, and – 5 missions. All experiments were approved by the Institutional Animal Care and Use Committee of JAXA (Protocol Number: 016-014B for MHU-1, No. 018-011D for MHU-4, and No. 018-036D for MHU-5), Explora Biolabs (Study Number: EB15-010A for MHU-1, No. EB19-003 for MHU-4, and No. SP19-003 for MHU-5), and NASA (Protocol Number: NAS-15-004-Y1 for MHU-1, No. FLT-18-118 for MHU-4, and No. JAXA MHU-5/FLT-19-121 for MHU-5). All experiments were conducted according to the guidelines and applicable laws in Japan and the USA.

### MHU missions

Figure 1 show an overview of the three missions.

MHU-1: Twelve male C57BL/6 J mice (8 weeks old) in the TCU were launched aboard SpX-9 on June 18, 2016, from the NASA Kennedy Space Center (KSC) in Florida and were then transported to the ISS. The mice were divided into two groups: the MG group experienced microgravity, and the AG group was exposed to 1 *g* artificial gravity on the bottom floor of habitat cage units (HCUs) via centrifugation at 77 rpm in MARS-S (15 cm radius).

MHU-4: Six male C57BL/6 J mice (9 weeks old) in the TCU were launched aboard SpX-17 on May 7, 2019, from the NASA KSC and were then transported to the ISS. The MHU-4_PG group was maintained in an artificial gravity environment at 1/6 *g* on the bottom floor of the HCU via centrifugation at 31 rpm in MARS-S (15 cm radius).

MHU-5: Six C57BL/6 J male mice (9 weeks old) in the TCU were launched aboard SpX-20 on March 10, 2020, from the NASA KSC and were then transported to the ISS. The MHU-5_PG group was maintained in an artificial gravity environment at 1/6 *g* on the bottom floor of the HCU via centrifugation at 21 rpm in MARS-L (35 cm radius).

The GC group comprised six age-matched (8 or 9 weeks old) male C57BL/6 J mice per mission, maintained under the same conditions as those aboard the ISS. See Supplementary Information 1 for a detailed description of the space flight experiments. All mice from each mission were sacrificed on the second day after returning to Earth.

### Sample collection and preparation

The left and right Sol and EDL muscles were neatly stacked on top of each other. The Achilles tendon side was vertically mounted on the tragacanth gum on a cork disc and quickly frozen in isopentane cooled in liquid nitrogen. Thin frozen muscle sections (8 µm) were subjected to immunohistochemical and RNA-seq analyses.

### RNA-seq analysis

Using TRIzol (Thermo Fisher Scientific, Waltham, MA, USA), total RNA was extracted from 100 sections of 8 μm frozen Sol muscle tissue, following standard protocols. An RNA-seq library was prepared using the NEBNext Ultra II Directional RNA Library Prep Kit (New England Biolabs, Ipswich, MA, USA) after ribosomal RNA (rRNA) depletion using the NEBNext rRNA Depletion Kit (New England Biolabs). Paired-end (2 × 36 bases) sequencing was performed using the NextSeq500 platform (Illumina, San Diego, CA, USA). FASTQ files were imported to the CLC Genomics Workbench (version 10.1.1; Qiagen, Hilden, Germany). Sequence reads were mapped to the mouse reference genome (mm10). Gene expression was calculated as total read counts normalized by transcripts per million. Genes with 0 counts in any sample were excluded, and differential expression was analyzed using the ‘empirical analysis of DGE’ tool (edgeR test) in CLC Main Workbench (version 21.0.3; Qiagen). Differentially expressed genes were extracted among conditions (MHU-1_GC vs. MHU-1_MG, MHU-4_GC vs. MHU-4_PG, and MHU-5_GC vs. MHU-5_PG) with FDR-corrected *P* < 0.05. A PCA plot was constructed using Python.

### Gene functional analysis

Clustering heatmap was generated in Morpheus (https://software.broadinstitute.org/morpheus/). GO analysis of each cluster was performed using the Enrichr webtool^56–58^. GSEA was conducted according to published methods^59,60^.

### Histological and immunohistochemical analyses of muscle cryosections

Frozen tissue sections (8 μm in thickness) were mounted on glass slides and subjected to HE staining and immunohistochemical analysis. For immunohistochemical analysis, the sections were air-dried, fixed at −20 °C with acetone, washed with phosphate-buffered saline (PBS), blocked with 5% goat serum/1% bovine serum albumin/PBS and M.O.M. blocking reagent (Vector Laboratories) for 1 h at temperature range. After this, the sections were incubated with primary antibodies and M.O.M. protein concentrate (Vector Laboratories) overnight at 4 °C. The primary antibodies used were mouse monoclonal antibody (BA-D5, 1:50) against myosin heavy chain 7 (Myh7) for type I fibers, mouse:monoclonal antibody (SC-71, 1:100) against Myh2 for type IIa fibers, and mouse monoclonal antibody (BF-F8, 1:100) against myosin heavy chain 4 (Myh4) for type IIb fibers. The primary antibodies were obtained from the Developmental Studies Hybridoma Bank (University of Iowa, Iowa City, IA, USA). After primary antibody incubation, all samples were washed and incubated with appropriate Alexa Fluor-conjugated secondary antibodies (Thermo Fisher Scientific, 1:1000) for 1 h at temperature range. Tissue sections were then mounted with VECTASHIELD Vibrance Antifade Mounting Medium (Vector Laboratories). The proportion of each myofiber type and the CSA of myofibers were assessed using a BIOREVO BZ-X800 microscope system and a hybrid cell count application (Keyence, Osaka, Japan). All myofibers were counted in the Sol and EDL muscle samples, for 279–2093 myofibers per section.

### Statistics and reproducibility

All data are represented as the mean of biological replicates and dots of individual samples data. Comparisons between groups (excluding RNA-seq analysis, as explained before) were performed using one-way ANOVA followed by the Tukey’s test. *P* < 0.05 was considered to indicate significance.

### Reporting summary

Further information on research design is available in the Nature Portfolio Reporting Summary linked to this article.

## Supplementary information

Supplementary Information

Description of Additional Supplementary Files

Supplementary Data 1

Supplementary Data 2

Supplementary Movie 1

Reporting Summary

## Data Availability

All data that support the findings of this study are available from the corresponding author upon reasonable request. Source data of figures are summarized in “Supplementary Data 1”. RNA-seq data are deposited in the DNA Databank of Japan (https://ddbj.nig.ac.jp/resource/sra-submission/DRA014378).

## Code availability

Python code for PCA plots and R code for dotplots for GO analysis are summarized in “Supplementary Data 2”.
