# Supplementary material for: Lunar gravity prevents skeletal muscle atrophy but not myofiber type shift in mice
Source: Commun Biol. 2023 Apr 21;6:424. doi: 10.1038/s42003-023-04769-3 (PMC10121599; doi:10.1038/s42003-023-04769-3)
Supplement: Supplementary file 1 — Supplementary Information [file 42003_2023_4769_MOESM1_ESM.pdf]

Research Article

Lunar gravity prevents skeletal muscle atrophy but not myofiber type shift in mice

Takuto Hayashi[1,2], Ryo Fujita[3], Risa Okada[4], Michito Hamada[1,5], Riku Suzuki[1,6], Sayaka Fuseya[1], James Leckey[1], Maho Kanai[1,6], Yuri Inoue[1,2], Shunya Sadaki[1,7], Ayano Nakamura[1,8], Yui Okamura[1,8], Chikara Abe[9], Hironobu Morita[5,10], Tatsuya Aiba[4], Teruhiro Senkoji[4], Michihiko Shimomura[4,5], Maki Okada[4], Daisuke Kamimura[4], Akane Yumoto[4,5], Masafumi Muratani[5,11] Takashi Kudo[1,5*], Dai Shiba[4,5*], Satoru Takahashi[1,5*]

[1]Laboratory Animal Resource Center in Transborder Medical Research Center, and Department of Anatomy and Embryology, Institute of Medicine, University of Tsukuba, Ibaraki 305-8575, Japan

[2]Doctoral Program in Biomedical Sciences, Graduate School of Comprehensive Human Sciences, University of Tsukuba, Ibaraki 305-8575, Japan

[3]Divsion of Regenerative Medicine, Transborder Medical Research Center, Institute of Medicine, University of Tsukuba, Ibaraki 305-8575, Japan

[4]JEM Utilization Center, Human Spaceflight Technology Directorate, Japan Aerospace Exploration Agency (JAXA), Ibaraki 305-8505, Japan

[5]Mouse Epigenetics Project, ISS/Kibo experiment, JAXA, Ibaraki 305-8505, Japan

[6]Ph.D. Program in Human Biology, School of Integrative and Global Majors, University of Tsukuba, Ibaraki 305-8575, Japan

[7]Ph.D. Program in Humanics, School of Integrative and Global Majors, University of Tsukuba, Ibaraki 305-8575, Japan

[8]College of Medicine, School of Medicine and Health Sciences, University of Tsukuba, Ibaraki 305-8575, Japan

[9]Department of Physiology, Gifu University Graduate School of Medicine, Gifu, 501-1194, Japan

[10]Department of Nutrition Management, Tokai Gakuin University, Gifu, 504-8511, Japan

[11]Department of Genome Biology, Transborder Medical Research Center, Institute of Medicine, University of Tsukuba, Ibaraki 305-8575, Japan

Supplementary information

Return phase and animal dissection

After unberthing from the ISS, the Dragon vehicle splashed down in the Pacific Ocean near California. After a ship picked up the cargo, the returned TCU was transported to a port in Long Beach. JAXA later received the TCU from NASA and transported it to Explora Biolabs in San Diego for analysis. The health condition of the mice was evaluated, and their body weights measured. The mid-air righting reflex test was conducted. Briefly, each mouse oriented in the spine position (back to the ground) was dropped from a height of about 40 cm above a padded surface. This trial was repeated 3–5 times per mouse, and the average time for righting was analyzed from a high speed (480 fps) video (DSC-RX100M5, SONY, Tokyo, Japan). The rotarod performance test was conducted after the righting reflex test. Rotation speed was increased from 2 to 40 rpm in 2 min (47600, Bioresearch Center, Nagoya, Japan), and the time each mouse spent on the rotating rod was measured. Each mouse was subjected to three rotarod tests. Isoflurane-anesthetized mice were euthanized by exsanguination and then dissected to collect tissue samples.

Environmental parameters in the flight missions

During the flight, temperature and humidity data loggers were attached to the TCU (launch and return phase) or inside the centrifuge-equipped biological experiment facility (CBEF-ALT) or CBEF-L (onboard phase) in all three missions. Following launch of the MHU-4 and MHU-5 missions, the logger data were processed; these data are summarized in Supplementary Tables 1 and 2. The MHU-1 mission data were previously published[28].

The average temperatures at the onboard phases of MHU-4 and MHU-5 were 23.1 ºC and 23.6 ˚C, respectively. Temperatures higher than 30 ˚C were observed in the return phase of MHU-5, especially in the sea transportation phase, and lasted 16 h. The average relative humidities at the onboard phases were 47.5% and 39.7% for MHU-4 and MHU-5, respectively. The gas concentrations of carbon dioxide and ammonia were monitored by sensors attached inside the CBEF-ALT or CBEF-L. These concentrations were kept at a low level during the mission (Supplementary Tables 1 and 2). The results indicate that mice were housed under a properly controlled environment.

Since the radiation level in space is much higher than that on the ground, the total radiation dose during the approximately one-month flight was measured using the Bio Passive Dosimeter for Life Science Experiments in Space (Bio PADLES). The radiation absorbed dose rate was 0.31 ± 0.02 (MHU-4) and 0.36 ± 0.02 (MHU-5) mGy/day, and the dose equivalent rate was 0.48 ± 0.03 (MHU-4) and 0.79 ± 0.05 (MHU-5) mSv/day

during the two space experiments. While high doses of radiation affect the health of mice, these values were comparable to those of the MHU-1 mission and appeared to be low-level, not causing the acute reactions in mice as those observed in astronauts.

Habitation data from the flight experiments (MHU-4 and MHU-5) and hardware malfunctions during the return phase of MHU-5

As no electronic scales were on board of the ISS, it was not possible to measure mice body weight nor their consumption of food and water. All six mice in MHU-4 and three mice in MHU-5 either gained weight or maintained their pre-flight weight, but three mice in MHU-5 significantly lost weight compared to their pre-flight values (Supplementary Fig. 1a, b). We estimated the water consumption onboard from video analysis during the weekly food bar change operation by the astronauts (Supplementary Fig. 1c) and found no differences between mice in MHU-5, suggesting the return phase as the cause for the body weight loss. The ground test using the TCU used for live animal return from the ISS reproduced this body weight loss of mice reared in cages that the weight loss was observed (cage# A15, A19, and A21; Supplementary Fig. 2). The TCU has a water supply system mainly composed of two water balloons and a drink nozzle, which supplies water when mice tilt the stem bar of the drink nozzle[28]. The stem bar can be moved at 360 ^{o}; however, we found that water flow from the drink valves, even from a new nozzle, was not comparable in every direction. The TCU has cylindrical cages[28], and it was observed that the water nozzles from the TCU cages where the mice that lost weight were housed had a similar characteristic: water did not easily come out when the stem bar was tilted toward the back of cage. In contrast, in the cages of the mice that maintained or gained weight, this was not observed; that is, these mice could access the water nozzle from every direction (Supplementary Fig. 2c). These results suggest that this directional property of the water nozzle is important when mice are reared in the TCU because of its cylindrical shape that would restrict access directions to the water nozzle. After the ground tests, the water nozzles were disassembled, and no anomaly or clog was found inside the drinking valves. For future MHU missions, we will screen out the water supply system that might prevent water access. From these results, we concluded that the body weight loss of the three mice in MHU-5 was due to the malfunction of the water supply system of the TCU during the return phase, and therefore, we excluded the three mice from subsequent analyses. The change in mean body weight pre-launch and post-landing was recalculated excluding the three mice that lost weight in MHU-5. For both missions, the rate of body weight change in PG mice was significantly smaller than that in GC mice (Supplementary Fig. 1c).

Onboard operations by the ISS crew and estimation of food/water consumption

Mice were transported to the ISS using TCU by SpX-17 (MHU-4) and SpX-20 (MHU-5). After the Dragon vehicle of SpX berthed with the ISS, mice were transferred to the HCU by crew members. Mouse-husbandry tasks included exchanging food cartridges, supplying water, collecting waste, and replacing odor filters. The provided food (approximately 35 *g*) was contained in a cartridge, supporting one-week habitation. The cartridges had windows and scales at their sides, allowing the estimation of the remaining amount of food. This estimation was performed via video at the weekly food cartridge exchange operations. Water was replenished once a week by the crew, and the volumes were measured. After about one-month habitation onboard, the mice were transferred to the TCU for the return to Earth.

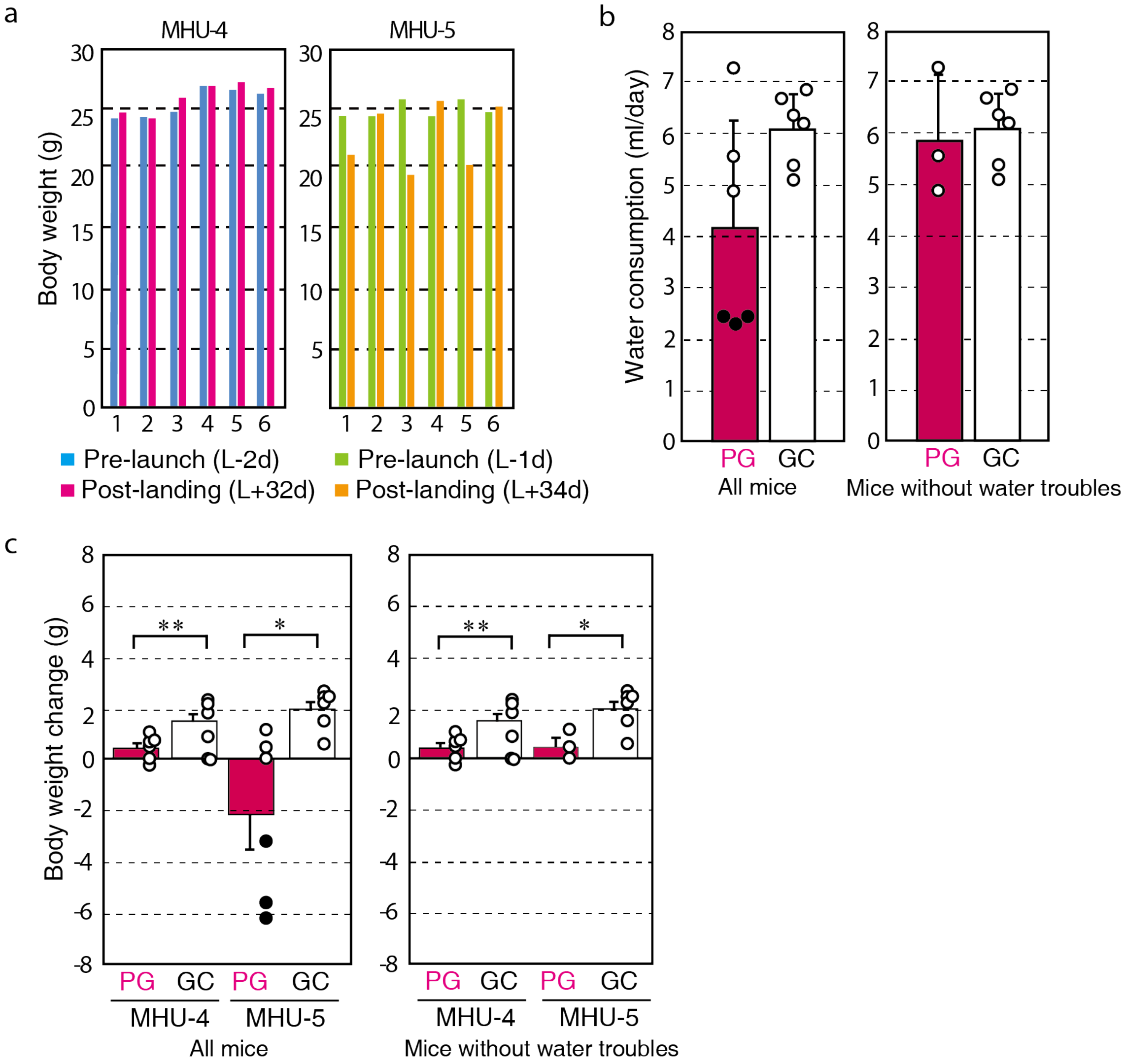

Supplementary Figure 1. Weight change of each individual before and after launch

a, Summarized data of body weight (g) pre-launch and post-landing.

b, Overall water consumption onboard and during the return phase. The water consumption data were recalculated for all mice excluding ID nos. 1, 3, and 5 in the mouse habitat unit (MHU)-5 mission.

c, Body weight change between pre-launch and post-landing. Body weight data were recalculated for all mice excluding ID nos. 1, 3, and 5 in the MHU-5 mission. Data are represented as the mean ± standard error of the mean. *P < 0.05 and **P < 0.01, as determined using the Student's *t*-test.

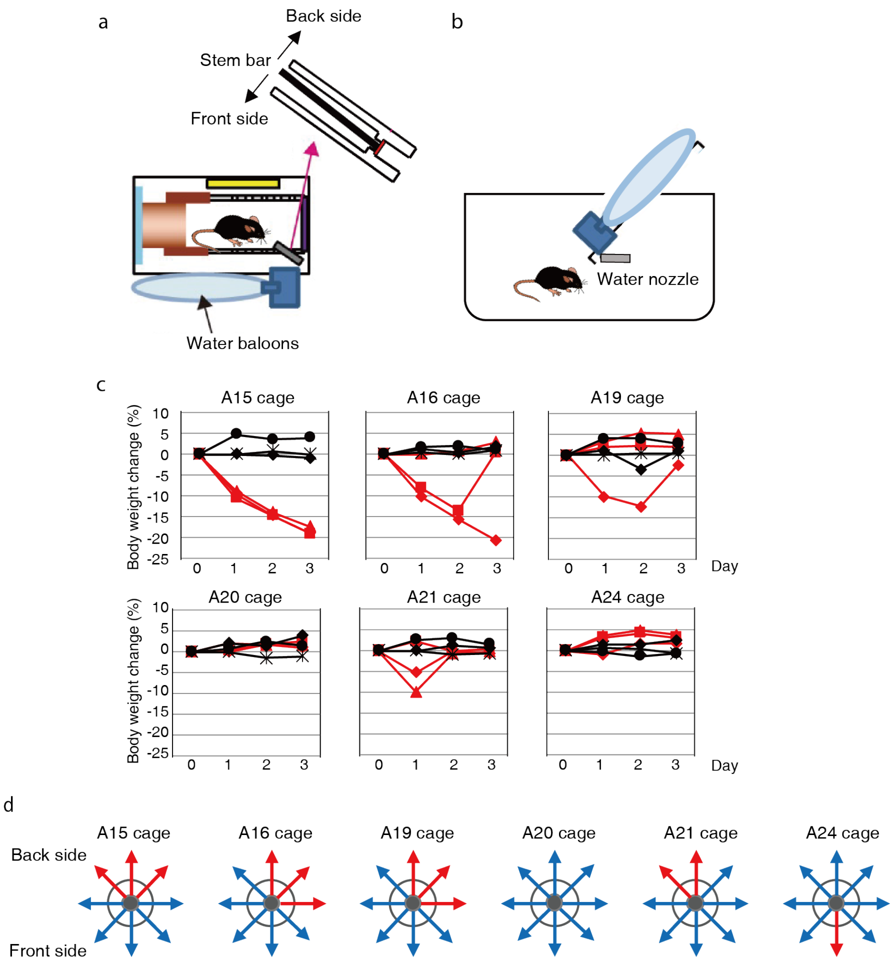

Supplementary Figure 2. Association between water nozzles and body weight change

a, Schematic outline of the ground test using the transportation cage unit (TCU) returned from the International Space Station (ISS). The water nozzle is attached to water balloons for water supply upon movement of the stem bar. The stem bar can be moved 360º.

b, Schematic outline of the ground test using a vivarium cage attached to the water supply system of the TCU returned from the ISS.

c, Three mice were maintained for 3 d in the cages with the configuration illustrated in a (red lines) or b (black lines). Change in body weight relative to the body weight on day 0 is shown. Each line indicates an individual mouse.

d, Schematic diagrams of the water nozzle tip. The circle and center dot indicate the seat and stem bar of the water nozzle, respectively. The stem bar was moved in eight directions, as indicated by the arrows. The 12 o'clock position represents the stem bar movement toward the back side of the TCU. Red arrows indicate no or little water flow from the water nozzle, and blue arrows represent proper water flow.

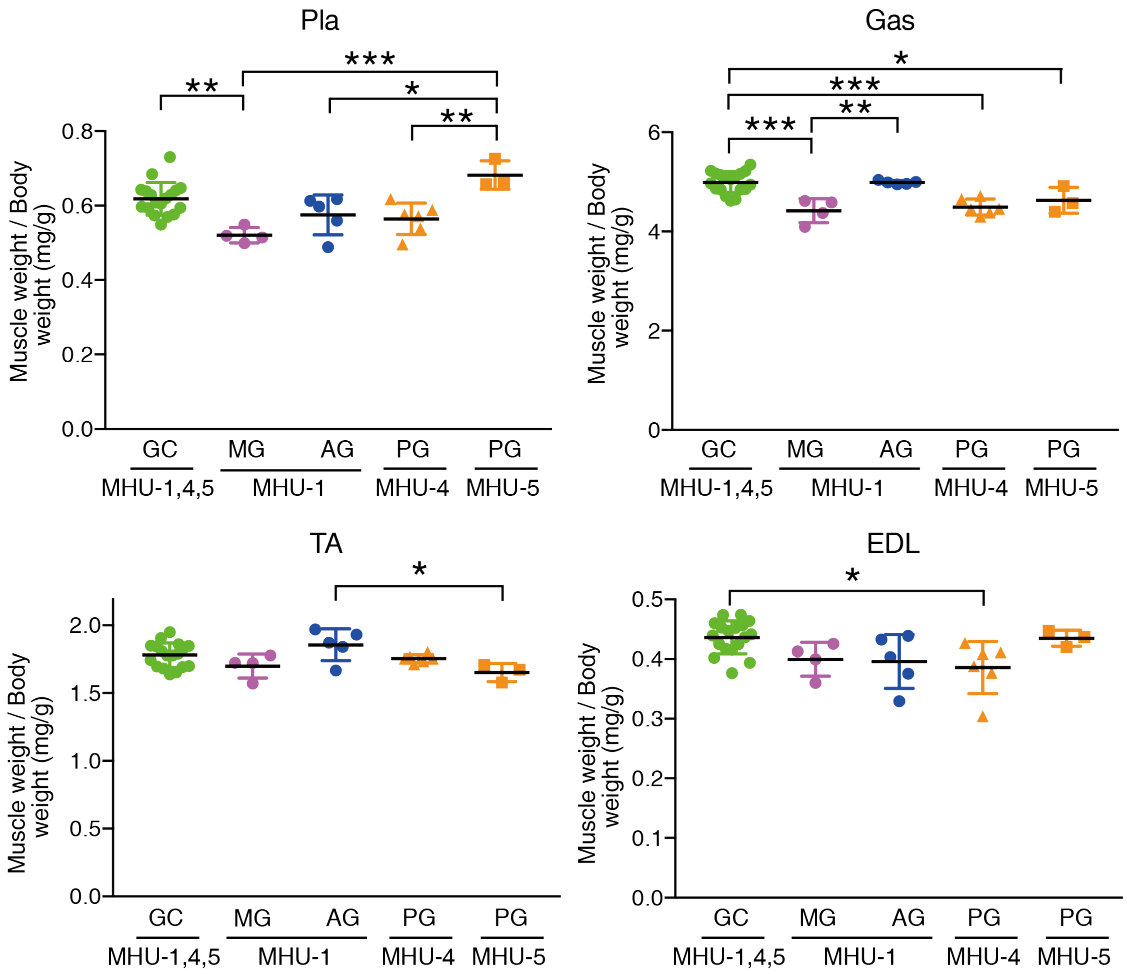

Supplementary Figure 3. Effect of lunar gravity on skeletal muscle weight

Skeletal muscle weights normalized to the body weight in each group (*n* = 3). *n* = 9 for GC. *P < 0.05, **P < 0.01, ***P < 0.001, as determined using Tukey's test. Data are represented as mean, standard deviation (SD), and each point represents an individual mouse.

Pla: plantaris; Gas: gastrocnemius; TA: tibialis anterior; EDL: extensor digitorum longus; GC: ground control; MG: microgravity; PG: partial gravity [1/6 *g*]; AG: artificial gravity [1 *g*]; MHU: mouse habitat unit.

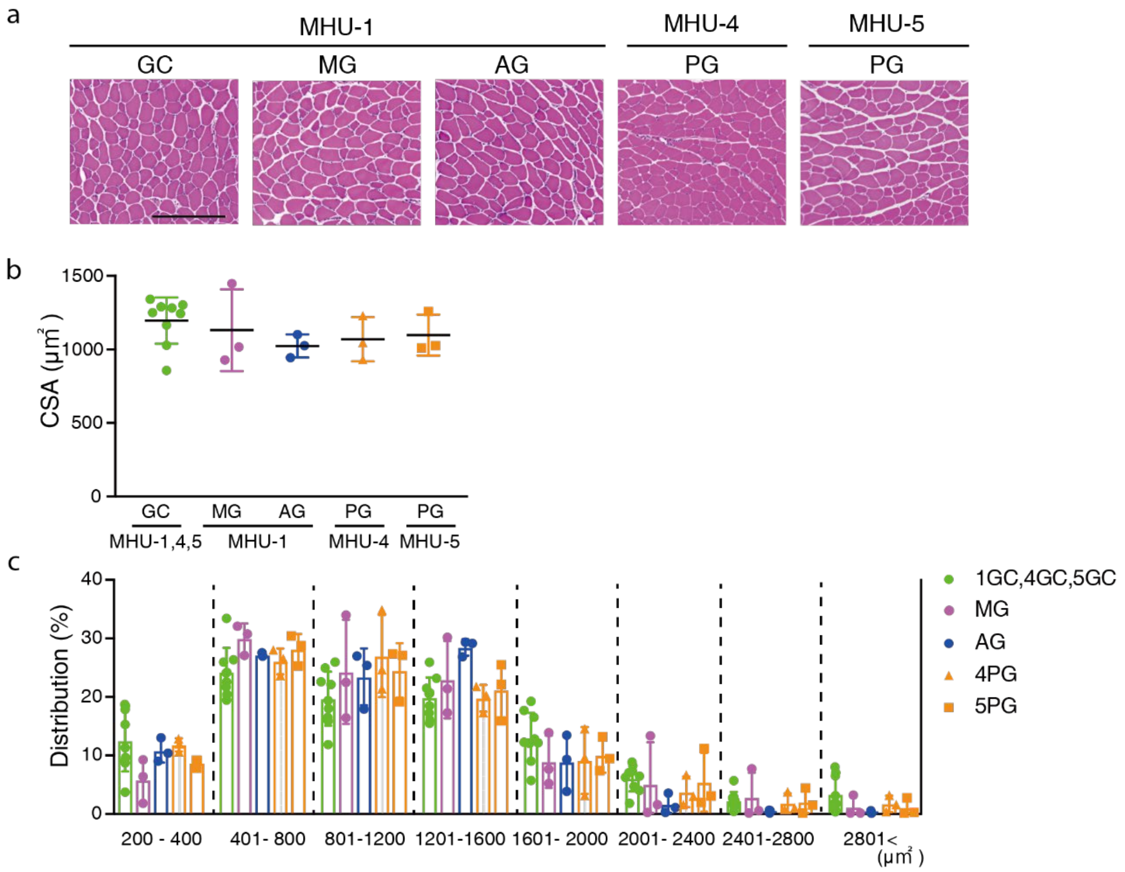

Supplementary Figure 4. Effect of lunar gravity on the extensor digitorum longus (EDL) muscle

a, Hematoxylin–eosin staining of EDL muscle cross sections. Scale bar, 200 μm.

b, Cross-sectional area (CSA) of EDL myofibers in each group. $n = 9$ for GC and $n = 3$ for all other groups. Data are represented as mean, SD, each point represents an individual mouse.

c, CSA distribution of EDL myofibers in each group. $n = 9$ for GC and $n = 3$ for all other groups. Data are represented as mean, SD, and each point represents an individual mouse.

1GC: MHU-1_GC; 4GC: MHU-4_GC; 5GC: MHU-5_GC; MG: MHU-1_MG; AG: MHU-1_AG; 4PG: MHU-4_PG; 5PG: MHU-5_PG

GC: ground control; MG: microgravity; PG: partial gravity [1/6 *g*]; AG: artificial gravity [1 *g*]; MHU: mouse habitat unit.

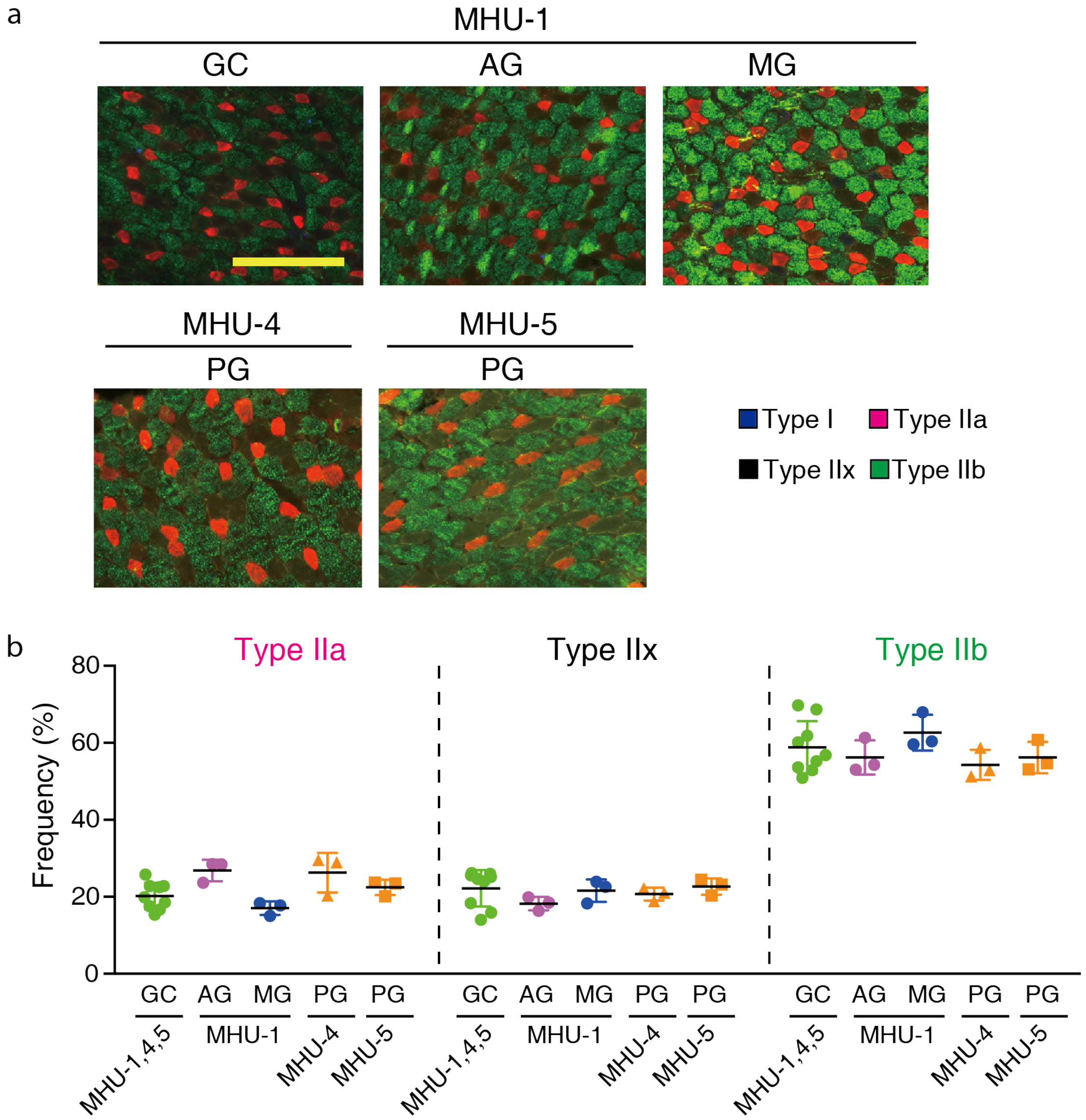

Supplementary Figure 5. Effect of lunar gravity on myofiber type in the extensor digitorum longus (EDL) muscle

a, Immunohistochemical staining for myosin heavy chain using BA-D5 (type I; blue), SC-71 (type IIa; red), and BF-F3 (type IIb; green) antibodies. Unstained myofibers are assumed to be type IIx (black). Scale bar, 200 μm.

b, Frequency of respective myofiber types in the EDL muscle for each group. *n* = 9 for GC and *n* = 3 for all other groups. Data are represented as mean, SD and each point represents an individual mouse.

Supplementary Table 1.
Environmental parameters in mouse habitat unit (MHU)-4

Items	Data source	Launch	On-orbit	Return	Remarks
Temperature (˚C)	CBEF-ALT	N/A	21.4-28.1 (AVE 23.1)	N/A	
	TCU	23.0-29.0 (AVE 25.1)	N/A	22.5-27.5 (AVE 24.0)	
	HCU telemetry	N/A	20.2-26.8 (AVE 23.5)	N/A	
Humidity (Relative humidity; %)	CBEF-ALT	N/A	32.0-58.0 (AVE 47.5)	N/A	
	TCU	21.9-56.1 (AVE 30.2)	N/A	26.8-52.5 (AVE 35.1)	Less than 30%: 32 h for lunch, 19.7 h for return
Carbon dioxide concentration (%)	HCU telemetry	N/A	0.20-0.39 (AVE 0.31)	N/A	
Ammonia concentration (ppm)	HCU telemetry	N/A	0.1-1.0 (AVE 0.36)	N/A	

CBEF-ALT; Centrifuge-equipped biological experiment facility-ALT
TCU; Transportation cage unit
HCU; Habitat cage unit

Supplementary Table 2.
Environmental parameters in mouse habitat unit (MHU)-5

Items	Data source	Launch	On-orbit	Return	Remarks
Temperature (˚C)	CBEF-L	N/A	22.3-24.6 (AVE 23.6)	N/A	
	TCU	22.5-29.0 (AVE 27.2)	N/A	20-32.5 (AVE 28.4)	Higher than 30˚C: 16 h for return, during sea transportation
	HCU telemetry	N/A	20.9-26.0 (AVE 22.6)	N/A	
Humidity (Relative humidity; %)	CBEF-L	N/A	36-43.7 (AVE 39.7)	N/A	
	TCU	21.3-58.7 (AVE 27.7)	N/A	21.8-47.0 (AVE 30.0)	Less than 30%: 46 h for launch, 44.7 h for return
Carbon dioxide concentration (%)	HCU telemetry	N/A	0.16-0.24 (AVE 0.19)	N/A	
Ammonia concentration (ppm)	HCU telemetry	N/A	0-1.0 (AVE 0.14)	N/A	

CBEF-L; Centrifuge-equipped biological experiment facility-L

TCU; Transportation cage unit

HCU; Habitat cage unit

Supplementary Movie 1.

Onboard habitation of mouse habitat unit (MHU)-1, MHU-4, and MHU-5 mice at the midpoint of the mission (day 16) (10 s).

Top panel: MHU-1 mice subjected to microgravity; second panel from the top: MHU-4_PG mice; third panel from the top: MHU-5_PG mice; bottom panel: MHU-1 mice subjected to 1 *g* artificial gravity.
