# Supplementary material for: Lunar gravity prevents skeletal muscle atrophy but not myofiber type shift in mice
Source: Commun Biol. 2023 Apr 21;6:424. doi: 10.1038/s42003-023-04769-3 (PMC10121599; doi:10.1038/s42003-023-04769-3)
Supplement: Supplementary file 2 — Description of Additional Supplementary Files [file 42003_2023_4769_MOESM2_ESM.pdf]

Description of Additional Supplementary Files

File name: Supplementary Data 1

Description: The source data behind the graphs in the paper

File name: Supplementary Data 2

Description: The Python and R code which are used in the paper figures

File name: Supplementary Movie 1

Description: Onboard habitation of mouse habitat unit (MHU)-1, MHU-4, and MHU-5 mice at the midpoint of the mission (day 16) (10 s). Top panel: MHU-1 mice subjected to microgravity; second panel from the top: MHU4_PG mice; third panel from the top: MHU-5_PG mice; bottom panel: MHU-1 mice subjected to 1 g artificial gravity.
