# Supplementary material for: Lunar gravity prevents skeletal muscle atrophy but not myofiber type shift in mice
Source: Commun Biol. 2023 Apr 21;6:424. doi: 10.1038/s42003-023-04769-3 (PMC10121599; doi:10.1038/s42003-023-04769-3)
Supplement: Supplementary file 4 — Supplementary Data 2 [file 42003_2023_4769_MOESM4_ESM.pdf]

**Research Article**

# Lunar gravity prevents skeletal muscle atrophy but not myofiber type shift in mice

Takuto Hayashi[1,2], Ryo Fujita[3], Risa Okada[4], Michito Hamada[1,5], Riku Suzuki[1,6], Sayaka Fuseya[1], James Leckey[1], Maho Kanai[1,6], Yuri Inoue[1,2], Shunya Sadaki[1,7], Ayano Nakamura[1,8], Yui Okamura[1,8], Chikara Abe[9], Hironobu Morita[5,10], Tatsuya Aiba[4], Teruhiro Senkoji[4], Michihiko Shimomura[4,5], Maki Okada[4], Daisuke Kamimura[4], Akane Yumoto[4,5], Masafumi Muratani[5,11] Takashi Kudo[1,5*], Dai Shiba[4,5*], Satoru Takahashi[1,5*]

[1]Laboratory Animal Resource Center in Transborder Medical Research Center, and Department of Anatomy and Embryology, Faculty of Medicine, University of Tsukuba, Ibaraki 305-8575, Japan
[2]Doctoral Program in Biomedical Sciences, Graduate School of Comprehensive Human Sciences, University of Tsukuba, Ibaraki 305-8575, Japan
[3]Divsion of Regenerative Medicine, Transborder Medical Research Center, Faculty of Medicine, University of Tsukuba, Ibaraki 305-8575, Japan
[4]JEM Utilization Center, Human Spaceflight Technology Directorate, Japan Aerospace Exploration Agency (JAXA), Ibaraki 305-8505, Japan
[5]Mouse Epigenetics Project, ISS/Kibo experiment, JAXA, Ibaraki 305-8505, Japan
[6]Ph.D. Program in Human Biology, School of Integrative and Global Majors, University of Tsukuba, Ibaraki 305-8575, Japan
[7]Ph.D. Program in Humanics, School of Integrative and Global Majors, University of Tsukuba, Ibaraki 305-8575, Japan
[8]College of Medicine, School of Medicine and Health Sciences, University of Tsukuba, Ibaraki 305-8575, Japan
[9]Department of Physiology, Gifu University Graduate School of Medicine, Gifu, 501-1194, Japan
[10]Department of Nutrition Management, Tokai Gakuin University, Gifu, 504-8511, Japan
[11]Department of Genome Biology, Transborder Medical Research Center, Faculty of Medicine, University of Tsukuba, Ibaraki 305-8575, Japan

**Code of Python for PCA plots.**

```
import math
import numpy as np
import pandas as pd
import matplotlib.pyplot as plt
from sklearn.decomposition import PCA
from sklearn.preprocessing import scale

df = pd.read_excel(".xlsx",index_col=0)
Expression = df.iloc[:, df.columns.str.contains('Normalized')].astype(float)
print(Expression.shape)
print(Expression.max())
Expression.head()

Expression = Expression.sort_index(axis=1)
Expression.head()
print(Expression.max())

df_count_tpm = Expression.copy()
tpm = scale(df_count_tpm.T)
pca = PCA(n_components=3)
pca.fit(tpm)

principalComponents = pca.fit_transform(tpm)
principalDf = pd.DataFrame(data = principalComponents
                , columns = ['PC 1', 'PC 2', 'PC 3'], index=df_count_tpm.T.index)
principalDf["target"] = ['4-PG','4-PG','4-PG','4-GC','4-GC','4-GC',
                         '5-PG','5-PG','5-PG','5-GC','5-GC','5-GC',
                        '1-AG','1-AG','1-AG','1-MG','1-MG','1-MG','1-GC','1-GC','1-
GC']
principalDf.head(len(principalDf))
```

```
print( pca.components_.round(4))
print( pca.mean_)
print(pca.get_covariance())
print(pca.explained_variance_ratio_)
print(sum(pca.explained_variance_ratio_))
print([math.sqrt(u) for u in pca.explained_variance_])
fuka = (pca.components_*np.c_[np.sqrt(pca.explained_variance_)]).T
print(fuka.round(4))


plt.style.use('default')
fig = plt.figure(figsize = (5,5))
ax = fig.add_subplot(1,1,1)
ax.set_xlabel('PC 1', fontsize = 15)
ax.set_ylabel('PC 2', fontsize = 15)

ax.set_title('2 component PCA', fontsize = 20)
for target, color in zip(targets,colors):
    indicesToKeep = principalDf['target'] == target
    ax.scatter(principalDf.loc[indicesToKeep, 'PC 1']
               , principalDf.loc[indicesToKeep, 'PC 2']
               , c = color
               , s = 50)

ax.legend(targets,bbox_to_anchor=(1.7, 1), fontsize=13)
ax.grid()
plt.show()
```

**Code of R for dotplots of GO analysis results.**

```
library(ReactomePA)

sample_data<-read.csv("c4.csv", header=T, fileEncoding="utf-8")

Enrichment <- sample_data$Padj
library(ggplot2)
Enrichment <- -log10(sample_data$Padj)
p = ggplot(sample_data, aes(x = Ratio, y = reorder(Term, Enrichment)))
p = p + geom_point()
p = p + geom_point(aes(size = Counts)) + labs (x = "Ratio", y = "Term")
pbubble = p + geom_point(aes(size = Counts, color = Enrichment))
pr = pbubble + scale_color_gradient(low = "purple", high = "orange")
pr
```
